# Force and Torque Model of Magnetically Levitated System with 2D Halbach Array and Printed Circuit Board Coils

**DOI:** 10.3390/s23218735

**Published:** 2023-10-26

**Authors:** Menglong Zou, Mingxing Song, Shun Zhou, Xianze Xu, Fengqiu Xu

**Affiliations:** School of Electrical Information, Wuhan University, Wuhan 430072, China; zoumenglong@whu.edu.cn (M.Z.); songmx@whu.edu.cn (M.S.); zhoushun1997@whu.edu.cn (S.Z.)

**Keywords:** planar motor, motion decoupling, numerical magnetic force and torque model

## Abstract

Precision machining fields often require worktables with different stroke sizes. To address the need for scalability and facilitate manufacturing, this study proposes a novel infinite expansion magnetically levitated planar motor (MLPM) based on PCB stator coils. Different from existing magnetic levitation systems that use PCB coils, the design presented in this paper utilizes smaller coil units, with each coil being independent of one another. The coils are structured in a spiral pattern on a 16-layer PCB, comprising 15 layers of coils, while the last layer is dedicated to wiring and other circuits. Magnetic field modeling is conducted for both the stator coil and the 2D Halbach array structure employed in the system. A simple table lookup method is employed to accurately account for the prevalent end effects observed during system motion. Additionally, the decoupling effect of magnetic force and torque is evaluated by solving for the current vector at different points along a specific trajectory. To verify the accuracy of the proposed system’s modeling, a prototype is developed and tested. Experimental results demonstrate that compared to traditional harmonic model methods, the proposed approach improves the calculation accuracy of magnetic force by 50.31% and torque by 70.65%. This study presents a new MLPM system with vast potential applications in precision manufacturing and robotics. The innovative design and improved performance characteristics make it a promising technology for enhancing the capabilities of worktables in precision machining fields.

## 1. Introduction

Magnetic levitation technology has garnered significant interest in various applications, including chip manufacturing [1,2,3,4], microscopic imaging [5,6,7,8], and force control [9,10,11,12]. Its advantages of being friction-less [13], non-polluting [14], and having high precision capabilities [15] make it highly desirable. Presently, multiple processing methods, such as extreme ultraviolet lithography [16,17,18], require workbenches to carry workpieces between several stations. Long-stroke movements are necessary for these applications. While long-stroke movements have been studied in the literature [19,20,21], the excitation coils used in these systems are typically constructed using traditional copper wire. However, these coils require a significant amount of material and are challenging to manufacture, leading to high costs when extending the working stroke. To enhance the performance and manufacturability of magnetic levitation motors, several researchers have explored the use of printed circuit boards (PCBs) for constructing stator coils [22,23,24].

The utilization of printed circuit boards (PCBs) in magnetic levitation technology offers potential benefits such as reduced volume and higher current density, resulting in enhanced levitation and propulsion capabilities. A direct drive 6-DOF moving magnet levitation planar motor is proposed in [22]. The motor features a 1D Halbach array and stationary coils constructed using a printed circuit board. The literature also discusses the mitigation of force pulsation in magnetic levitation planar motors by employing appropriately segmented and spaced magnet arrays. This development in magnetic levitation planar motors shows promise for various industrial automation and manufacturing applications. A prototype of a magnetic levitation planar motor with the same magnet array and coil structure is created in [23]. However, this system incorporates a fixed-position laser displacement sensor to detect the translator’s position, leading to limitations in its working stroke. The research highlights the need for further improvements in position detection mechanisms. In a different approach, a novel magnetic levitation system with a PCB-based coil structure is described in [24]. This system employs hall sensors for accurate position measurement and demonstrates high precision. While the system exhibits scalability, it is worth noting that the coil size is large, and all coils are concentrated on a single PCB, posing practical challenges for expansion.

**Figure 1:**
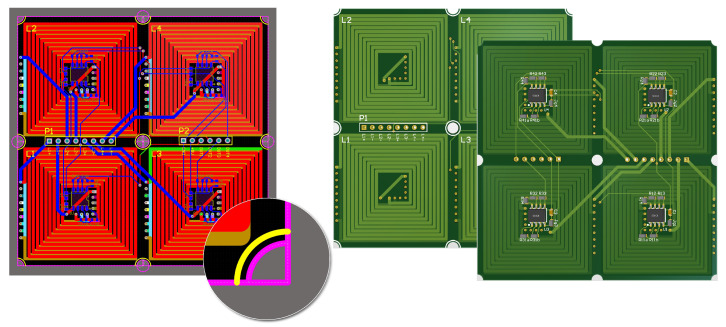
PCB integrated with four coils and hall sensors.

This paper introduces a novel infinite expansion magnetically levitated planar motor (MPLM) with moving magnets. The coils are strategically arranged in a spiral pattern on a 16-layer PCB, as shown in Figure 1. This PCB comprises 15 layers dedicated to the coils, while the remaining layer is reserved for wiring and other essential circuits. Each PCB integrates four independent coils, each with its own power supply and drive mechanism. The PCBs are designed to be spliced together, enabling easy extension of the working stroke by attaching a new PCB at the stator’s edge. Furthermore, the four coils on each PCB can operate independently without interfering with other existing components. To accurately detect the position of the translator, four hall sensors are integrated on each PCB. The translator itself consists of a 2D Halbach array embedded in an aluminum plate. A loading platform, which is created using 3D printing technology, is positioned above the aluminum plate. This innovative design and construction allow for infinite expansion possibilities and provide efficient and reliable operation for the MLPM.

**Figure 2:**
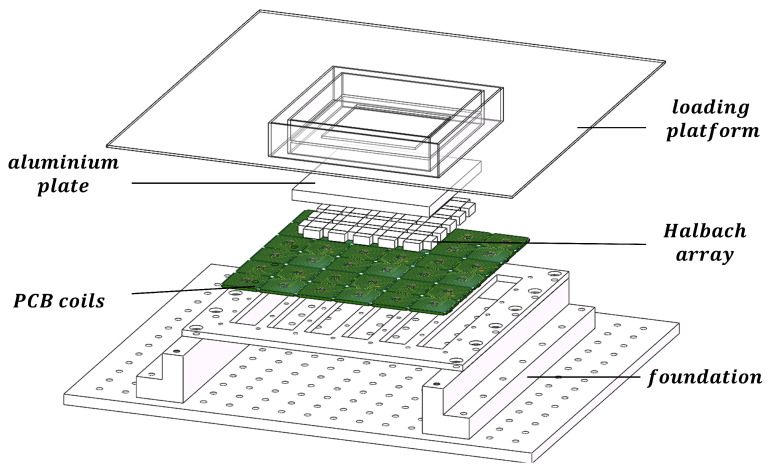
Overview of proposed magnetically levitated planar motor.

For the magnetic levitation system structure designed above, this article first conducts magnetic field modeling. The MLPM given in Figure 2 is employed as the study object to introduce the numerical model and commutation method. In order to calculate the magnetic force and torque, the existing harmonic models have been developed and are well established in the literature, as reported in [25]. The magnetic flux density of the 2D Halbach array is expressed as a Fourier series in this literature. On this basis, the paper puts forward an analytical model for determining the forces and torque of the proposed structure. To minimize the significant error traditionally generated by harmonic models, a simple table lookup method is used to fit end effects and is added to the new model. The new analytical model offers high accuracy and lower computational costs compared with the existing methods. Moreover, this paper presents a real-time calculation method for the commutation process that converts the position coordinates of the translator into the current vector. This approach enables the magnetic levitation worktable to meet its real-time control requirements.

Section 2 introduces the novel infinite expansion magnetically levitated planar motor proposed. The calculation and analysis of force and torque are presented in Section 3. In Section 4, the paper proposes a commutation method by decoupling the force and torque. To verify the accuracy and effectiveness of the proposed method, Section 5 compares the calculation results with the results of measurement and the commercial software Radia (version 4.1). Finally, Section 6 concludes the paper.

## 2. Structure of The Magnetically Levitated Planar System

MLPM possesses unique inherent advantages for high-precision manufacturing and processing applications. Its desirable features, including an isolation environment, multiple degrees of freedom, and strong carrying capacity, render it applicable in specialized high-precision equipment such as extreme ultraviolet lithography machines. With the advancement of MLPM technology, there is a growing demand for higher working stroke capabilities. To address this need, an innovative MLPM system with infinite expansion capability is proposed in this article. In pursuit of a simpler structure and extended working stroke, the design of the proposed structure is delineated in Figure 2. This study offers a solution to enhance the performance of MLPM for high-precision manufacturing and processing applications.

**Figure 3:**
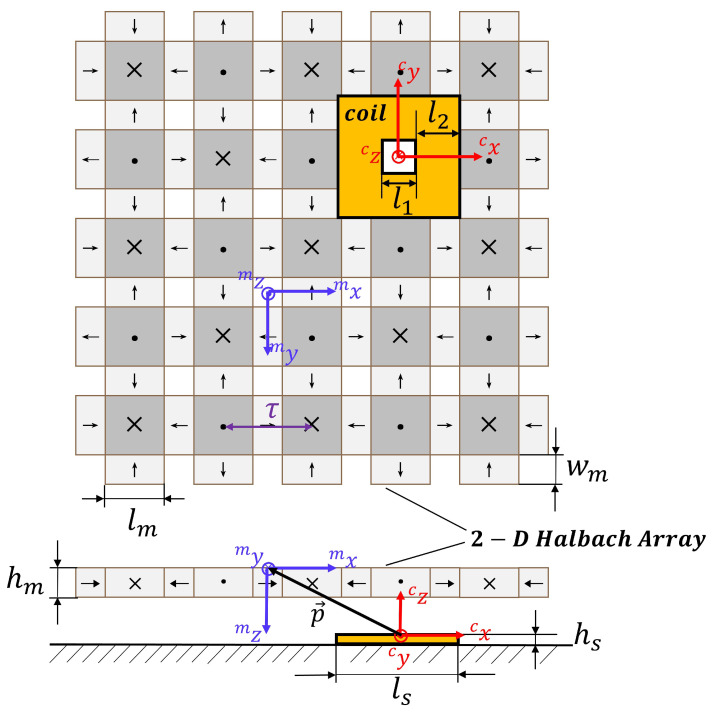
Bottom and elevation view of the components of 2D Halbach array and a coil.

The key components of the MLPM proposed in this article are the stator and translator, responsible for generating the magnetic force. The stator comprises spliceable PCBs that integrate both hall sensors and coils. The coil design employed in this system resembles a hollow square shape, featuring a hollow side length denoted as $l_{1}$ and a single side coil width of $l_{2}$. On the other hand, the translator adopts a 2D Halbach array structure, which ensures a unilateral magnetic field strength to enhance levitation and propulsion effects. There are two different sizes in the magnet array—the large one and the small one. Each magnet in the array has dimensions of $l_{m}$, $w_{m}$ and $h_{m}$. The large magnetic blocks are characterized by equal length and width, while the long side of a small magnet matches the long side of a large magnet, and the short side corresponds to its height. The magnetization direction of each magnet is depicted in Figure 3. These magnets are embedded within an aluminum plate and united as a single unit. Aluminum, known for its lightweight properties and high strength, serves as the ideal material for supporting the translator. The primary design principle throughout the entire system aims to achieve a simple structure capable of infinite expansion of the working plan.

## 3. Analytical Model of Force and Torque

Two coordinate systems have been established to analyze the force and torque between coil and magnets given in Figure 3. The vectors ${}^{c}x={\left({}^{c}x{,}^{c}y{,}^{c}z\right)}^{T}$ and ${}^{m}x={\left({}^{m}x{,}^{m}y{,}^{m}z\right)}^{T}$ represent the coordinates of any point in the global and local coordinate system, respectively. Additionally, the vector $p={\left({}^{c}p_{x}{,}^{c}p_{y}{,}^{c}p_{z}\right)}^{T}$ describes the relative translation, while the vector $q={\left(\alpha ,\beta ,\theta \right)}^{T}$ denotes the relative rotation between the global coordinate system and local coordinate system. The design sizes of the coil and 2D Halbach array are also depicted in this figure and given by $l_{s}$, $l_{1}$, $l_{2}$, $h_{s}$, $l_{m}$, $w_{m}$, $h_{m}$ and τ.

Based on the arrangement principle of the Halbach array and the position of the coordinate origin, the magnetic flux density at any point ${}^{m}x={\left({}^{m}x{,}^{m}y{,}^{m}z\right)}^{T}$ can be expressed by Equation (1). The calculation procedure for obtaining this magnetic flux density has been highly studied in the existing literature [25].

(1) $$\begin{array}{c} {}^{m}B\left({}^{m}x\right)=e^{\lambda^{m}z}\left[\begin{array}{c} B_{xy}\cos\left(\frac{\pi^{m}x}{\tau }\right)\sin\left(\frac{\pi^{m}y}{\tau }\right)\\ B_{xy}\sin\left(\frac{\pi^{m}x}{\tau }\right)\cos\left(\frac{\pi^{m}y}{\tau }\right)\\ B_{z}\sin\left(\frac{\pi^{m}x}{\tau }\right)\sin\left(\frac{\pi^{m}y}{\tau }\right) \end{array}\right], \end{array}$$

where $B_{xy}$ and $B_{z}$ are the coefficients representing the effective amplitudes of the first harmonic of the flux density distribution. The parameter λ is defined as

(2) $$\begin{array}{c} \lambda =\frac{\sqrt{2}\pi }{\tau }, \end{array}$$

where τ denotes the pole pitch, as also shown in Figure 3.

**Figure 4:**
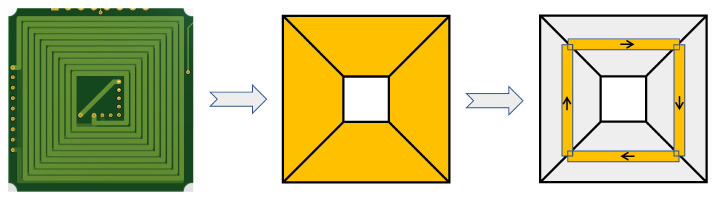
Four individual segments of a coil.

In order to accommodate the restrictions imposed by the operational speed of the controller, it becomes imperative to simplify the force and torque model of the coil. In this particular model, the current area of the square is divided into four individual segments, as illustrated in Figure 4. Each segment of the straight part is represented as a current segment positioned along the centerline. It is considered infinitely thin and possesses a length of $l_{1}+l_{2}$. The disparity between this simplified model and the actual measured values is merely 3.2% to 5.7%. This level of error can be regarded as negligible within acceptable limits.

The Lorentz integral is employed to determine the magnetic force and torque acting on the translator. The coil is divided into four individual segments, for which the integral region is the summation of these segments. The mass center point of the coil is assumed as ${}^{m}x_{s}={\left({}^{m}x_{s},{}^{m}y_{s},{}^{m}z_{s}\right)}^{T}$, while the computed force and torque are expressed as ${}^{m}F={\left({}^{m}F_{x},{}^{m}F_{y},{}^{m}F_{z}\right)}^{T}$ and ${}^{m}T={\left({}^{m}T_{x},{}^{m}T_{y},{}^{m}T_{z}\right)}^{T}$, respectively. Assuming the current through the coil is *I* in Ampere-turns, the expressions for force and torque integrals, calculated using the local coordinate system, are presented as follows.

(3) $$\begin{array}{ccc} {}^{m}F & = & -\sum_{i=1}^{4}\int_{C}{}^{m}J\times {}^{m}Bdl\\ & = & -\int_{{}^{m}x_{s}-\frac{l_{1}+l_{2}}{2}}^{{}^{m}x_{s}+\frac{l_{1}+l_{2}}{2}}{\left[\begin{array}{ccc} I & 0 & 0 \end{array}\right]}^{T}\times {}^{m}B{\left(x^{\prime },{}^{m}y_{s}-\frac{l_{1}+l_{2}}{2},{}^{m}z_{s}\right)}^{T}dx^{\prime }\\ & & -\int_{{}^{m}y_{s}-\frac{l_{1}+l_{2}}{2}}^{{}^{m}y_{s}+\frac{l_{1}+l_{2}}{2}}{\left[\begin{array}{ccc} 0 & I & 0 \end{array}\right]}^{T}\times {}^{m}B{\left({}^{m}x_{s}+\frac{l_{1}+l_{2}}{2},y^{\prime },{}^{m}z_{s}\right)}^{T}dy^{\prime }\\ & & -\int_{{}^{m}x_{s}-\frac{l_{1}+l_{2}}{2}}^{{}^{m}x_{s}+\frac{l_{1}+l_{2}}{2}}{\left[\begin{array}{ccc} -I & 0 & 0 \end{array}\right]}^{T}\times {}^{m}B{\left(x^{\prime },{}^{m}y_{s}+\frac{l_{1}+l_{2}}{2},{}^{m}z_{s}\right)}^{T}dx^{\prime }\\ & & -\int_{{}^{m}y_{s}-\frac{l_{1}+l_{2}}{2}}^{{}^{m}y_{s}+\frac{l_{1}+l_{2}}{2}}{\left[\begin{array}{ccc} 0 & -I & 0 \end{array}\right]}^{T}\times {}^{m}B{\left({}^{m}x_{s}-\frac{l_{1}+l_{2}}{2},y^{\prime },{}^{m}z_{s}\right)}^{T}dy^{\prime } \end{array}$$

(4) $$\begin{array}{ccc} {}^{m}T & = & \int_{C}{}^{m}x_{s}\times {}^{m}J\times {}^{m}B\;dl\\ & = & {-}^{m}x_{s}\times {}^{m}F \end{array}$$

where ${}^{m}J$, ${}^{m}B$ and p represent the current density inside the coil, the magnetic flux density created by the 2D Halbach array and the relative translation vector, respectively. According to the vector product algorithm of vectors, it can be found that

(5) $$\begin{array}{ccc} {}^{m}F_{x} & = & -\int_{{}^{m}y_{s}-\frac{l_{1}+l_{2}}{2}}^{{}^{m}y_{s}+\frac{l_{1}+l_{2}}{2}}I\;\cdot \;e^{-\lambda^{m}z_{s}}B_{z}\sin\left(\frac{\pi \left({}^{m}x_{s}+\frac{l_{1}+l_{2}}{2}\right)}{\tau }\right)\sin\left(\frac{\pi y^{\prime }}{\tau }\right)dy^{\prime }\\ & & +\int_{{}^{m}y_{s}-\frac{l_{1}+l_{2}}{2}}^{{}^{m}y_{s}+\frac{l_{1}+l_{2}}{2}}I\;\cdot \;e^{-\lambda^{m}z_{s}}B_{z}\sin\left(\frac{\pi \left({}^{m}x_{s}-\frac{l_{1}+l_{2}}{2}\right)}{\tau }\right)\sin\left(\frac{\pi y^{\prime }}{\tau }\right)dy^{\prime }\\ & = & -2I\frac{\tau }{\pi }e^{-\lambda^{m}z_{s}}B_{z}\sin\left(\frac{\pi \left({}^{m}x_{s}+\frac{l_{1}+l_{2}}{2}\right)}{\tau }\right)\sin\left(\frac{\pi^{m}y_{s}}{\tau }\right)\sin\left(\frac{\pi \left(l_{1}+l_{2}\right)}{2\tau }\right)\\ & & +2I\frac{\tau }{\pi }e^{-\lambda^{m}z_{s}}B_{z}\sin\left(\frac{\pi \left({}^{m}x_{s}-\frac{l_{1}+l_{2}}{2}\right)}{\tau }\right)\sin\left(\frac{\pi^{m}y_{s}}{\tau }\right)\sin\left(\frac{\pi \left(l_{1}+l_{2}\right)}{2\tau }\right)\\ & = & -4I\frac{\tau }{\pi }e^{-\lambda^{m}z_{s}}B_{z}\sin^{2}\left(\frac{\pi \left(l_{1}+l_{2}\right)}{2\tau }\right)\cos\left(\frac{\pi^{m}x_{s}}{\tau }\right)\sin\left(\frac{\pi^{m}y_{s}}{\tau }\right) \end{array}$$

(6) $$\begin{array}{ccc} {}^{m}F_{y} & = & \int_{{}^{m}x_{s}-\frac{l_{1}+l_{2}}{2}}^{{}^{m}x_{s}+\frac{l_{1}+l_{2}}{2}}I\;\cdot \;e^{-\lambda^{m}z_{s}}B_{z}\sin\frac{\pi x^{\prime }}{\tau }\sin\left(\frac{\pi \left({}^{m}y_{s}-\frac{l_{1}+l_{2}}{2}\right)}{\tau }\right)dx^{\prime }\\ & & -\int_{{}^{m}x_{s}-\frac{l_{1}+l_{2}}{2}}^{{}^{m}x_{s}+\frac{l_{1}+l_{2}}{2}}I\;\cdot \;e^{-\lambda^{m}z_{s}}B_{z}\sin\left(\frac{\pi x^{\prime }}{\tau }\right)\sin\left(\frac{\pi \left({}^{m}y_{s}+\frac{l_{1}+l_{2}}{2}\right)}{\tau }\right)dx^{\prime }\\ & = & 2I\frac{\tau }{\pi }e^{-\lambda^{m}z_{s}}B_{z}\sin\left(\frac{\pi \left({}^{m}y_{s}-\frac{l_{1}+l_{2}}{2}\right)}{\tau }\right)\sin\left(\frac{\pi^{m}x_{s}}{\tau }\right)\sin\left(\frac{\pi \left(l_{1}+l_{2}\right)}{2\tau }\right)\\ & & -2I\frac{\tau }{\pi }e^{-\lambda^{m}z_{s}}B_{z}\sin\left(\frac{\pi \left({}^{m}y_{s}+\frac{l_{1}+l_{2}}{2}\right)}{\tau }\right)\sin\left(\frac{\pi^{m}x_{s}}{\tau }\right)\sin\left(\frac{\pi \left(l_{1}+l_{2}\right)}{2\tau }\right)\\ & = & -8I\frac{\tau }{\pi }e^{-\lambda^{m}z_{s}}B_{z}\sin\left(\frac{\pi \left(l_{1}+l_{2}\right)}{4\tau }\right)\sin\left(\frac{\pi^{m}x_{s}}{\tau }\right)\cos\left(\frac{\pi^{m}y_{s}}{\tau }\right) \end{array}$$

(7) $$\begin{array}{ccc} {}^{m}F_{z} & = & -\int_{{}^{m}x_{s}-\frac{l_{1}+l_{2}}{2}}^{{}^{m}x_{s}+\frac{l_{1}+l_{2}}{2}}I\;\cdot \;e^{-\lambda^{m}z_{s}}B_{xy}\sin\frac{\pi x^{\prime }}{\tau }\cos\left(\frac{\pi \left({}^{m}y_{s}-\frac{l_{1}+l_{2}}{2}\right)}{\tau }\right)dx^{\prime }\\ & & +\int_{{}^{m}y_{s}-\frac{l_{1}+l_{2}}{2}}^{{}^{m}y_{s}+\frac{l_{1}+l_{2}}{2}}I\;\cdot \;e^{-\lambda^{m}z_{s}}B_{xy}\cos\left(\frac{\pi \left({}^{m}x_{s}+\frac{l_{1}+l_{2}}{2}\right)}{\tau }\right)\sin\left(\frac{\pi y^{\prime }}{\tau }\right)dy^{\prime }\\ & & +\int_{{}^{m}x_{s}-\frac{l_{1}+l_{2}}{2}}^{m}I\;\cdot \;e^{-\lambda^{m}z_{s}}B_{xy}\sin\left(\frac{\pi x^{\prime }}{\tau }\right)\cos\left(\frac{\pi \left({}^{m}y_{s}+\frac{l_{1}+l_{2}}{2}\right)}{\tau }\right)dx^{\prime }\\ & & -\int_{{}_{m}y_{s}-\frac{l_{1}+l_{2}}{2}}^{m^{m}y_{s}+\frac{l_{1}+l_{2}}{2}}I\;\cdot \;e^{-\lambda^{m}z_{s}}B_{xy}\cos\left(\frac{\pi \left({}^{m}x_{s}-\frac{l_{1}+l_{2}}{2}\right)}{\tau }\right)\sin\left(\frac{\pi y^{\prime }}{\tau }\right)dy^{\prime }\\ & = & -2I\frac{\tau }{\pi }e^{-\lambda^{m}z_{s}}B_{xy}\cos\left(\frac{\pi \left({}^{m}y_{s}-\frac{l_{1}+l_{2}}{2}\right)}{\tau }\right)\sin\left(\frac{\pi^{m}x_{s}}{\tau }\right)\sin\left(\frac{\pi \left(l_{1}+l_{2}\right)}{2\tau }\right)\\ & & +2I\frac{\tau }{\pi }e^{-\lambda^{m}z_{s}}B_{xy}\cos\left(\frac{\pi \left({}^{m}x_{s}+\frac{l_{1}+l_{2}}{2}\right)}{\tau }\right)\sin\left(\frac{\pi^{m}y_{s}}{\tau }\right)\sin\left(\frac{\pi \left(l_{1}+l_{2}\right)}{2\tau }\right)\\ & & +2I\frac{\tau }{\pi }e^{-\lambda^{m}z_{s}}B_{xy}\cos\left(\frac{\pi \left({}^{m}y_{s}+\frac{l_{1}+l_{2}}{2}\right)}{\tau }\right)\sin\left(\frac{\pi^{m}x_{s}}{\tau }\right)\sin\left(\frac{\pi \left(l_{1}+l_{2}\right)}{2\tau }\right)\\ & & -2I\frac{\tau }{\pi }e^{-\lambda^{m}z_{s}}B_{xy}\cos\left(\frac{\pi \left({}^{m}x_{s}-\frac{l_{1}+l_{2}}{2}\right)}{\tau }\right)\sin\left(\frac{\pi^{m}y_{s}}{\tau }\right)\sin\left(\frac{\pi \left(l_{1}+l_{2}\right)}{2\tau }\right)\\ & = & -8I\frac{\tau }{\pi }e^{-\lambda^{m}z_{s}}B_{xy}\sin^{2}\left(\frac{\pi \left(l_{1}+l_{2}\right)}{2\tau }\right)\sin\left(\frac{\pi^{m}x_{s}}{\tau }\right)\sin\left(\frac{\pi^{m}y_{s}}{\tau }\right) \end{array}$$

The final calculation results of forces and moments can be expressed as

(8) $$\begin{array}{ccc} {}^{m}F & = & \left[\begin{array}{c} -4I\frac{\tau }{\pi }e^{-\lambda^{m}z_{s}}B_{z}\sin^{2}\left(\frac{\pi \left(l_{1}+l_{2}\right)}{2\tau }\right)\cos\left(\frac{\pi^{m}x_{s}}{\tau }\right)\sin\left(\frac{\pi^{m}y_{s}}{\tau }\right)\\ -8I\frac{\tau }{\pi }e^{-\lambda^{m}z_{s}}B_{z}\sin\left(\frac{\pi \left(l_{1}+l_{2}\right)}{4\tau }\right)\sin\left(\frac{\pi^{m}x_{s}}{\tau }\right)\cos\left(\frac{\pi^{m}y_{s}}{\tau }\right)\\ -8I\frac{\tau }{\pi }e^{-\lambda^{m}z_{s}}B_{xy}\sin^{2}\left(\frac{\pi \left(l_{1}+l_{2}\right)}{2\tau }\right)\sin\left(\frac{\pi^{m}x_{s}}{\tau }\right)\sin\left(\frac{\pi^{m}y_{s}}{\tau }\right) \end{array}\right] \end{array}$$

(9) $$\begin{array}{ccc} {}^{m}T & = & \left[\begin{array}{c} -{}^{m}y_{s}{}^{m}F_{z}+{}^{m}z_{s}{}^{m}F_{y}\\ -{}^{m}z_{s}{}^{m}F_{x}+{}^{m}x_{s}{}^{m}F_{z}\\ -{}^{m}x_{s}{}^{m}F_{y}+{}^{m}y_{s}{}^{m}F_{x} \end{array}\right] \end{array}$$

The aforementioned calculations are based on conventional harmonic models, which yield satisfactory magnetic fitting outcomes within the center region of the translator. However, considerable discrepancies arise when the coil is positioned at the edge region of the translator. In this system, a multitude of coils are situated at the periphery of the magnetic field at every translator position, thereby rendering the end effects an indispensable factor. Hence, it becomes imperative to account for the end effects in the proposed model.

**Figure 5:**
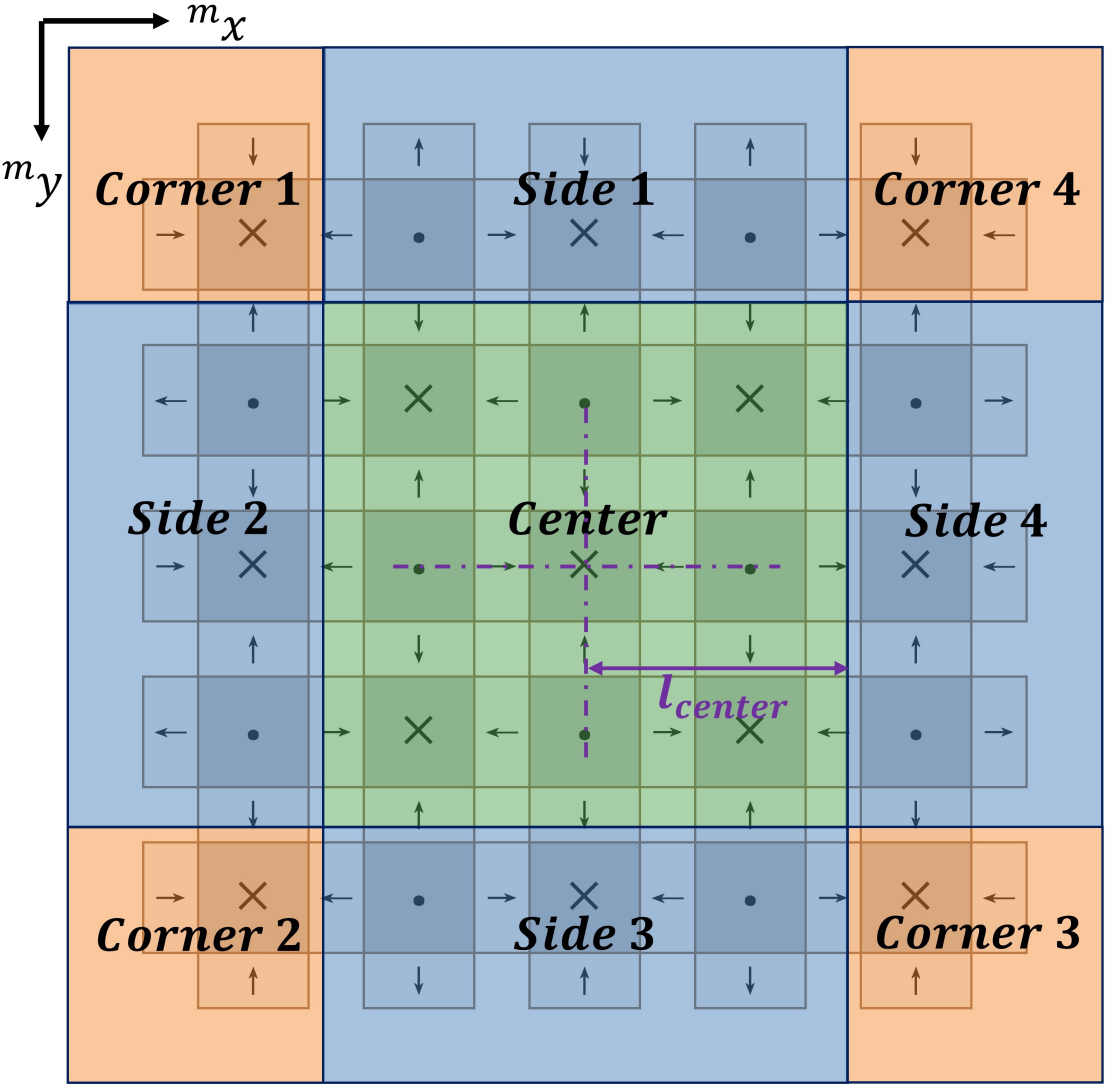
Division of magnetic field regions of translator.

In order to facilitate a more comprehensive analysis of the end effects, the 2D Halbach array employed in the system has been partitioned into three distinct regions: the center region, side region, and corner region, as illustrated in Figure 5. When the coil is positioned within the center region, the magnetic force exerted on the translator can be accurately predicted by employing traditional harmonic models, as calculated using Equation (8). On the other hand, the side and corner regions are further divided into four respective segments. Due to the rotational symmetry inherent in the magnet array, the calculation of magnetic force exhibits directional distinctions among different segments within each region.

According to the findings of end-effects research on a 2D Halbach array presented in [26], the magnetic force acting on the translator exhibits specific characteristics when the coil is situated in side region 4. These characteristics are outlined as follows:

1. Along the ${}^{m}y$ direction, the magnetic force distribution is in a sinusoidal shape, with the same phase as the center region, and the amplitude is changing. As the coil gradually moves away from the center region, the amplitude steadily diminishes until it approaches zero.
2. Along the ${}^{m}x$ direction, the magnetic force distribution exhibits a nonlinear wave shape. Each wave has a similar shape, but its amplitude is different.

The observed phenomenon can be attributed to the calculation of the magnetic field expression (1), which involves multiplying the Fourier series expressions along the ${}^{m}x$ and ${}^{m}y$ directions in the harmonic model. The resulting magnetic force expression (8) represents a superposition of components in both directions. In side region 4, the coil is situated close to the edge of the translator in the ${}^{m}x$ direction, leading to nonlinear attenuation of the magnetic field component in that direction. However, in the ${}^{m}y$ direction, the distance from the translator’s edge is relatively large, resulting in negligible decay of the magnetic field component. Consequently, the magnetic force along the ${}^{m}y$ direction continues to exhibit a sinusoidal shape. To address this nonlinear component, reference [26] proposes a complex function that fits it by measuring a large number of data points. However, due to the symmetrical design of the Halbach array used in this system, a single coefficient can be used to replace the complex function, significantly simplifying the computational workload and process. Based on these characteristics, the magnetic force in side region 4 can be corrected as follows:

(10) $$\begin{array}{c} {}^{m}F_{side\;4}=\left[\begin{array}{c} -4I\frac{\tau }{\pi }e^{-\lambda^{m}z_{s}}B_{z}\sin^{2}\left(\frac{\pi \left(l_{1}+l_{2}\right)}{2\tau }\right)k_{1}\sin\left(\frac{\pi^{m}y_{s}}{\tau }\right)\\ -8I\frac{\tau }{\pi }e^{-\lambda^{m}z_{s}}B_{z}\sin\left(\frac{\pi \left(l_{1}+l_{2}\right)}{4\tau }\right)k_{2}\cos\left(\frac{\pi^{m}y_{s}}{\tau }\right)\\ -8I\frac{\tau }{\pi }e^{-\lambda^{m}z_{s}}B_{xy}\sin^{2}\left(\frac{\pi \left(l_{1}+l_{2}\right)}{2\tau }\right)k_{3}\sin\left(\frac{\pi^{m}y_{s}}{\tau }\right) \end{array}\right] \end{array}$$

where $k_{1}$, $k_{2}$ and $k_{3}$ denote the coefficient of the nonlinear magnetic force components ${}^{m}F_{x}$, ${}^{m}F_{y}$ and ${}^{m}F_{z}$ in the ${}^{m}x$ direction within side region 4, respectively. These coefficients are dependent on the distance away from the center region along the direction of the force component, assuming that the height of the translator suspension remains constant. Likewise, the force equations for side regions 1, 2, and 3 can be defined as follows:

(11) $$\begin{array}{ccc} {}^{m}F_{side\;1} & = & \left[\begin{array}{c} -4I\frac{\tau }{\pi }e^{-\lambda^{m}z_{s}}B_{z}\sin^{2}\left(\frac{\pi \left(l_{1}+l_{2}\right)}{2\tau }\right)\cos\left(\frac{\pi^{m}x_{s}}{\tau }\right)k_{2}\\ -8I\frac{\tau }{\pi }e^{-\lambda^{m}z_{s}}B_{z}\sin\left(\frac{\pi \left(l_{1}+l_{2}\right)}{4\tau }\right)\sin\left(\frac{\pi^{m}x_{s}}{\tau }\right)\left(-k_{1}\right)\\ -8I\frac{\tau }{\pi }e^{-\lambda^{m}z_{s}}B_{xy}\sin^{2}\left(\frac{\pi \left(l_{1}+l_{2}\right)}{2\tau }\right)\sin\left(\frac{\pi^{m}x_{s}}{\tau }\right)k_{3} \end{array}\right] \end{array}$$

(12) $$\begin{array}{ccc} {}^{m}F_{side\;2} & = & \left[\begin{array}{c} -4I\frac{\tau }{\pi }e^{-\lambda^{m}z_{s}}B_{z}\sin^{2}\left(\frac{\pi \left(l_{1}+l_{2}\right)}{2\tau }\right)\left(-k_{1}\right)\sin\left(\frac{\pi^{m}y_{s}}{\tau }\right)\\ -8I\frac{\tau }{\pi }e^{-\lambda^{m}z_{s}}B_{z}\sin\left(\frac{\pi \left(l_{1}+l_{2}\right)}{4\tau }\right)\left(-k_{2}\right)\cos\left(\frac{\pi^{m}y_{s}}{\tau }\right)\\ -8I\frac{\tau }{\pi }e^{-\lambda^{m}z_{s}}B_{xy}\sin^{2}\left(\frac{\pi \left(l_{1}+l_{2}\right)}{2\tau }\right)k_{3}\sin\left(\frac{\pi^{m}y_{s}}{\tau }\right) \end{array}\right] \end{array}$$

(13) $$\begin{array}{ccc} {}^{m}F_{side\;3} & = & \left[\begin{array}{c} -4I\frac{\tau }{\pi }e^{-\lambda^{m}z_{s}}B_{z}\sin^{2}\left(\frac{\pi \left(l_{1}+l_{2}\right)}{2\tau }\right)\cos\left(\frac{\pi^{m}x_{s}}{\tau }\right)\left(-k_{2}\right)\\ -8I\frac{\tau }{\pi }e^{-\lambda^{m}z_{s}}B_{z}\sin\left(\frac{\pi \left(l_{1}+l_{2}\right)}{4\tau }\right)\sin\left(\frac{\pi^{m}x_{s}}{\tau }\right)k_{1}\\ -8I\frac{\tau }{\pi }e^{-\lambda^{m}z_{s}}B_{xy}\sin^{2}\left(\frac{\pi \left(l_{1}+l_{2}\right)}{2\tau }\right)\sin\left(\frac{\pi^{m}x_{s}}{\tau }\right)k_{3} \end{array}\right] \end{array}$$

Different from the side regions, the components in the ${}^{m}x$ and ${}^{m}y$ directions of the corner region will be nonlinear. Therefore, the force equations for the corner region involve two coefficients. The force equations for each corner are given by:

(14) $$\begin{array}{ccc} {}^{m}F_{corner\;1} & = & \left[\begin{array}{c} -4I\frac{\tau }{\pi }e^{-\lambda^{m}z_{s}}B_{z}\sin^{2}\left(\frac{\pi \left(l_{1}+l_{2}\right)}{2\tau }\right)\left(-k_{1}\right)k_{2}\\ -8I\frac{\tau }{\pi }e^{-\lambda^{m}z_{s}}B_{z}\sin\left(\frac{\pi \left(l_{1}+l_{2}\right)}{4\tau }\right)\left(-k_{2}\right)\left(-k_{1}\right)\\ -8I\frac{\tau }{\pi }e^{-\lambda^{m}z_{s}}B_{xy}\sin^{2}\left(\frac{\pi \left(l_{1}+l_{2}\right)}{2\tau }\right)k_{3}k_{3} \end{array}\right] \end{array}$$

(15) $$\begin{array}{ccc} {}^{m}F_{corner\;2} & = & \left[\begin{array}{c} -4I\frac{\tau }{\pi }e^{-\lambda^{m}z_{s}}B_{z}\sin^{2}\left(\frac{\pi \left(l_{1}+l_{2}\right)}{2\tau }\right)\left(-k_{1}\right)\left(-k_{2}\right)\\ -8I\frac{\tau }{\pi }e^{-\lambda^{m}z_{s}}B_{z}\sin\left(\frac{\pi \left(l_{1}+l_{2}\right)}{4\tau }\right)\left(-k_{2}\right)k_{1}\\ -8I\frac{\tau }{\pi }e^{-\lambda^{m}z_{s}}B_{xy}\sin^{2}\left(\frac{\pi \left(l_{1}+l_{2}\right)}{2\tau }\right)k_{3}k_{3} \end{array}\right] \end{array}$$

(16) $$\begin{array}{ccc} {}^{m}F_{corner\;3} & = & \left[\begin{array}{c} -4I\frac{\tau }{\pi }e^{-\lambda^{m}z_{s}}B_{z}\sin^{2}\left(\frac{\pi \left(l_{1}+l_{2}\right)}{2\tau }\right)k_{1}\left(-k_{2}\right)\\ -8I\frac{\tau }{\pi }e^{-\lambda^{m}z_{s}}B_{z}\sin\left(\frac{\pi \left(l_{1}+l_{2}\right)}{4\tau }\right)k_{2}k_{1}\\ -8I\frac{\tau }{\pi }e^{-\lambda^{m}z_{s}}B_{xy}\sin^{2}\left(\frac{\pi \left(l_{1}+l_{2}\right)}{2\tau }\right)k_{3}k_{3} \end{array}\right] \end{array}$$

(17) $$\begin{array}{ccc} {}^{m}F_{corner\;4} & = & \left[\begin{array}{c} -4I\frac{\tau }{\pi }e^{-\lambda^{m}z_{s}}B_{z}\sin^{2}\left(\frac{\pi \left(l_{1}+l_{2}\right)}{2\tau }\right)k_{1}k_{2}\\ -8I\frac{\tau }{\pi }e^{-\lambda^{m}z_{s}}B_{z}\sin\left(\frac{\pi \left(l_{1}+l_{2}\right)}{4\tau }\right)k_{2}\left(-k_{1}\right)\\ -8I\frac{\tau }{\pi }e^{-\lambda^{m}z_{s}}B_{xy}\sin^{2}\left(\frac{\pi \left(l_{1}+l_{2}\right)}{2\tau }\right)k_{3}k_{3} \end{array}\right] \end{array}$$

By utilizing a six-axis force sensor, the magnetic force in the corresponding coordinates can be measured, allowing for the determination of the coefficients $k_{1}$, $k_{2}$ and $k_{3}$. In this study, a total of 1500 data points are collected from side region 4 to validate the proposed model. The use of a 2D Halbach array, which exhibits symmetrical properties, enables a more efficient approach for handling end effects, requiring fewer data points. Specifically, when the suspension height remains constant, only one set of data needs to be measured on one side of the edge region, and the results can be applied to all other edge regions. This approach eliminates the need for least squares fitting of a function as employed in reference [26], resulting in a simplified data processing procedure and improved modeling performance.

According to [27], the controller necessitates the computation of force and torque applied to all coils at each sampling interval. Therefore, it is essential to develop expressions that are both efficient and straightforward. To achieve this objective, a segmented coil model is employed, along with three coefficients for compensating the magnetic errors arising from end effects. This approach enables the simultaneous consideration of calculation accuracy and efficiency. Consequently, the expressions for magnetic force and torque can be efficiently computed while maintaining an acceptable level of modeling accuracy. Furthermore, the simplicity of these expressions facilitates their implementation in practical control algorithms, which plays a crucial role in the success of any control system.

## 4. Commutation Method

To achieve precise and efficient motion control, only a subset of coils (denoted by *n*) below the magnet array are energized. By utilizing the magnetic force and torque models developed in the previous section, the force and torque exerted on the translator by a single active coil at various positions can be determined in a local coordinate system. These results can then be converted from the local coordinate system to the corresponding values in the global coordinate system, resulting in the derivation of the current–wrench transformation matrix represented in the global coordinate system. The current–wrench transformation matrix can be expressed as follows:

(18) $$\begin{array}{c} {}^{c}\Gamma =\left[\begin{array}{cccc} {}^{c}F_{x1} & {}^{c}F_{x2} & \cdots & {}^{c}F_{xn}\\ {}^{c}F_{y1} & {}^{c}F_{y2} & \cdots & {}^{c}F_{yn}\\ {}^{c}F_{z1} & {}^{c}F_{z2} & \cdots & {}^{c}F_{zn}\\ {}^{c}T_{x1} & {}^{c}T_{x2} & \cdots & {}^{c}T_{xn}\\ {}^{c}T_{y1} & {}^{c}T_{y2} & \cdots & {}^{c}T_{yn}\\ {}^{c}T_{z1} & {}^{c}T_{z2} & \cdots & {}^{c}T_{zn} \end{array}\right] \end{array}$$

By constructing the current–wrench transformation matrix using these equations, the resultants of force and torque from all *n* coils can be obtained through vector synthesis. Because the force is proportional to the current, the resultants of force and torque, which can be calculated by the formula of vector synthesis, applied by *n* coils, is

(19) $$\begin{array}{ccc} {}^{c}w & = & {\left[\begin{array}{cccccc} {}^{c}F_{x} & {}^{c}F_{y} & {}^{c}F_{z} & {}^{c}T_{x} & {}^{c}T_{y} & {}^{c}T_{z} \end{array}\right]}^{T}\\ & = & \left[\begin{array}{cccc} {}^{c}F_{x1} & {}^{c}F_{x2} & \cdots & {}^{c}F_{xn}\\ {}^{c}F_{y1} & {}^{c}F_{y2} & \cdots & {}^{c}F_{yn}\\ {}^{c}F_{z1} & {}^{c}F_{z2} & \cdots & {}^{c}F_{zn}\\ {}^{c}T_{x1} & {}^{c}T_{x2} & \cdots & {}^{c}T_{xn}\\ {}^{c}T_{y1} & {}^{c}T_{y2} & \cdots & {}^{c}T_{yn}\\ {}^{c}T_{z1} & {}^{c}T_{z2} & \cdots & {}^{c}T_{zn} \end{array}\right]\left[\begin{array}{c} I_{1}\\ I_{2}\\ \vdots \\ \vdots \\ I_{n-1}\\ I_{n} \end{array}\right] \end{array}$$

(20) $$\begin{array}{ccc} & = & {}^{c}\Gamma \;\cdot \;I, \end{array}$$

where I is the current vector containing the currents of *n* coils below the translator. To minimize power dissipation, namely the norm of the current vector, I is solved by

(21) $$\begin{array}{c} I={}^{c}\Gamma^{+}\;\cdot \;{}^{c}w={}^{c}\Gamma^{T}\;\cdot \;{\left({}^{c}\Gamma \;\cdot \;{}^{c}\Gamma^{T}\right)}^{-1}\;\cdot \;{}^{c}w, \end{array}$$

where ${}^{c}\Gamma^{+}$ is the pseudoinverse of the current–wrench transformation matrix ${}^{c}\Gamma$. With the sensor data obtained at each sampling cycle, the current–wrench transformation matrix ${}^{c}\Gamma$ is solved and then used to calculate the current vector I containing the exciting current for each coil.

**Figure 6:**
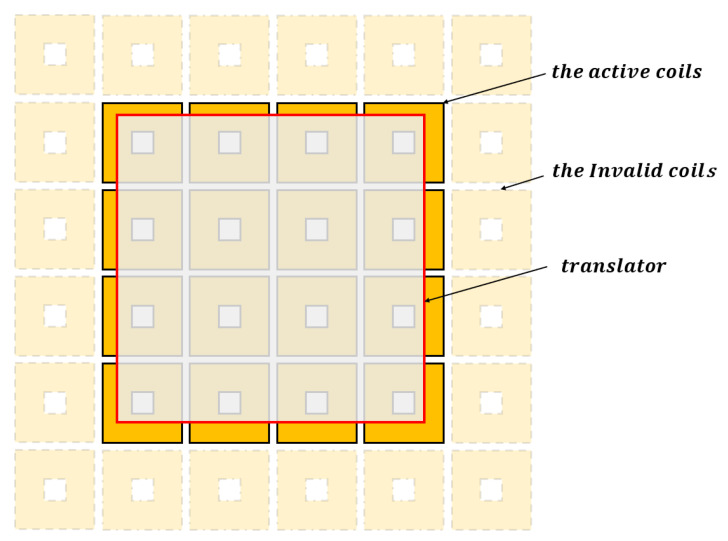
The coils below the translator.

For the MLPM given in Figure 6, parts of the coils under the translator should be actuated to produce the required force and torque. Specifically, the 16 coils located beneath the magnet array contribute the more significant force and torque compared with the coils far away from the magnet array. To validate the decoupling effect of the system, this study chooses to activate only these 16 coils positioned below the translator.

**Figure 7:**
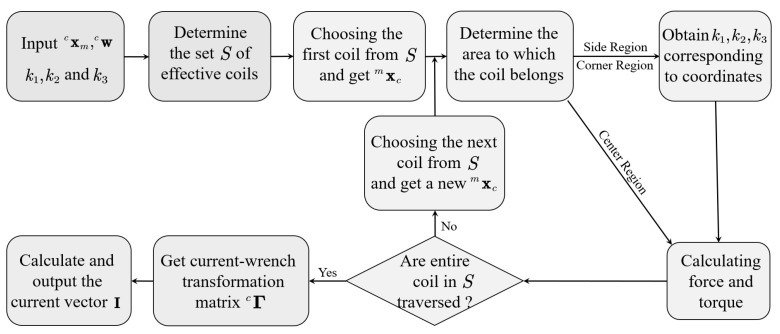
Force and torque computation on an offline program.

To facilitate the computation in the MLPM system, several steps are necessary. Firstly, the coefficients $k_{1}$, $k_{2}$ and $k_{3}$ corresponding to different distances between the coil and the center region need to be measured when the coil is positioned in the edge region at a specific height. These coefficients are then organized into a table, where each distance of the coil from the center region corresponds to a specific coefficient. Additionally, the position information of each coil from the physical model needs to be extracted and stored in memory. This information will be used in subsequent calculations. The interaction between all coils and the translator is solved using the method illustrated in Figure 7, which involves the following three main steps.

1. Based on the detected coordinate position of the translator using a hall sensor, the set *S* of conductive coils beneath the translator is determined. It is identified which coils need to be activated.
2. Starting from the first coil in set *S*, each coil’s position is evaluated one by one. The magnetic force and torque exerted by each coil on the translator are calculated until all coils have been traversed. If a coil is located in the center region, the force and torque can be computed directly using Equations (8) and (9). However, if a coil is positioned in the side or corner region, the corresponding coefficient is obtained from the lookup table based on the coil’s coordinates. This coefficient is then used to calculate the force and torque using the appropriate formula.
3. The current–wrench transformation matrix is derived, allowing the determination of the current vector using the method described in Equation (21). This enables precise control of the magnetic field generated by the coils, ensuring optimal system performance.

By following these three steps, an accurate model of the entire MLPM system can be established, and effective control can be achieved. The utilization of pre-stored position information and the current–wrench transformation matrix significantly reduces the computational burden. Moreover, the careful consideration of magnetic field interactions guarantees high-fidelity performance. Overall, this proposed method enables efficient and effective control of the magnetically levitated planar motor, delivering high precision with minimal power consumption.

## 5. Validation and Analysis

**Figure 8:**
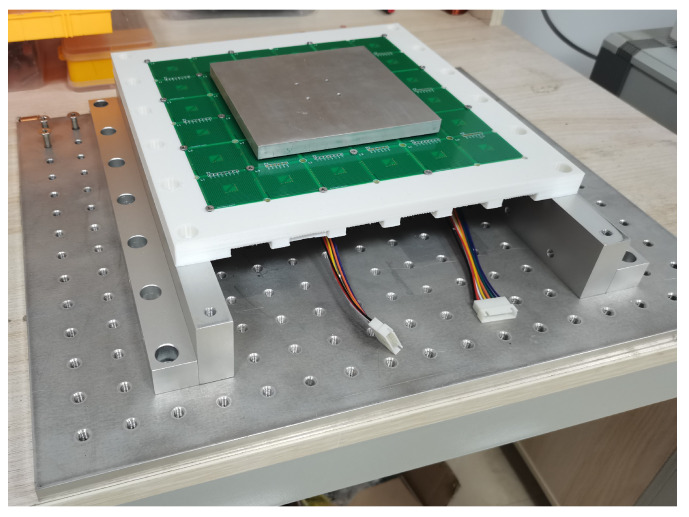
The principle prototype of MLPM including foundation, bracket, PCB coils, and translator.

**Figure 9:**
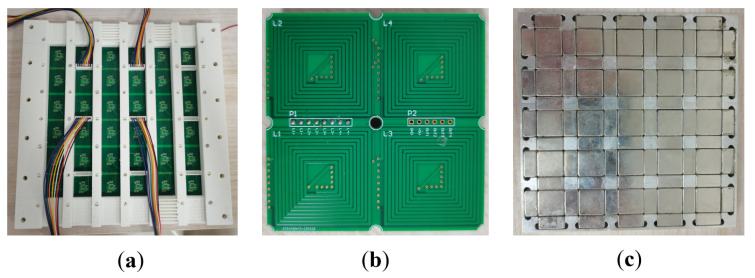
Three main components in the principle prototype (**a**) The bracket for fixing PCB coils. (**b**) The PCB coil is integrated with four independent exciting coils. (**c**) The bottom view of the translator.

To validate the accuracy of the proposed model and magnetic decoupling, a principle prototype is constructed for experimental purposes, as depicted in Figure 8. The foundation of the prototype is fabricated using an aluminum alloy, which provides stability and durability. Threaded holes are present on the foundation, facilitating easy fixation and adjustment of components. The PCB coil is mounted on a white bracket, which is fabricated using 3D printing technology and composed of PLA (polylactic acid), as shown in Figure 9a. This bracket serves as a support structure and allows precise positioning of the coil within the system. It is securely fixed onto the base of the prototype. The coil is positioned overhead, while the wiring is led out from beneath the system. This arrangement ensures a clean and organized workbench environment, minimizing any potential interference.

In the proposed system, the coils are arranged in a spiral pattern on a 16-layer PCB, as depicted in Figure 9b. The PCB is specifically designed to integrate 15 layers for housing the coils, with the remaining layer dedicated to wiring and other essential circuits. Each PCB incorporates four independent coils, each with its own power supply and drive mechanism. The size of a PCB that integrates four coils is 72 mm × 72 mm. The size of an individual coil is presented in Figure 3. The PCB design allows for easy extension of the working stroke by splicing together multiple PCBs at the stator’s edge. Moreover, due to the arrangement of coils on the PCB, the four coils can function independently without interfering with other existing components. This feature contributes to the reliability and flexibility of the system, providing a high degree of control and adjustability in various operational scenarios.

In the implemented prototype, a 2D Halbach array is integrated into an aluminum plate, serving as a translator as illustrated in Figure 9c. The translator has dimensions of 132 mm × 132 mm. During operation, the magnetic field of the system primarily concentrates on the lower surface. To address the end-effects phenomenon commonly observed during translator movement, the structure of the translator is designed to be symmetrical. Several indicator parameters of the prototype are presented in Table 1.

By constructing a prototype of the principle, the magnitude of the magnetic force acting on the rotor when the coil is located in the edge region can be measured, and the coefficient of the magnetic field can be calculated. It can also measure the electromagnetic force acting on the translator when a coil or multiple coils are energized with a specified current. In addition, the boundary element simulation software Radia is used for simulation experiments under the same conditions. The proposed model is compared to the commonly used harmonic model, Radia and measurement results. This comparison enables the quantification of the accuracy improvement achieved by utilizing the proposed model, which incorporates the non-linear effects existing in the system.

Through this verification process, the proposed model has been proven to have superior accuracy and fidelity compared to traditional harmonic models. This validation provides confidence in the proposed model’s ability to accurately predict the behavior of the planar motor, enabling effective design and control strategies.

### 5.1. Force and Torque of Proposed Model

**Figure 10:**
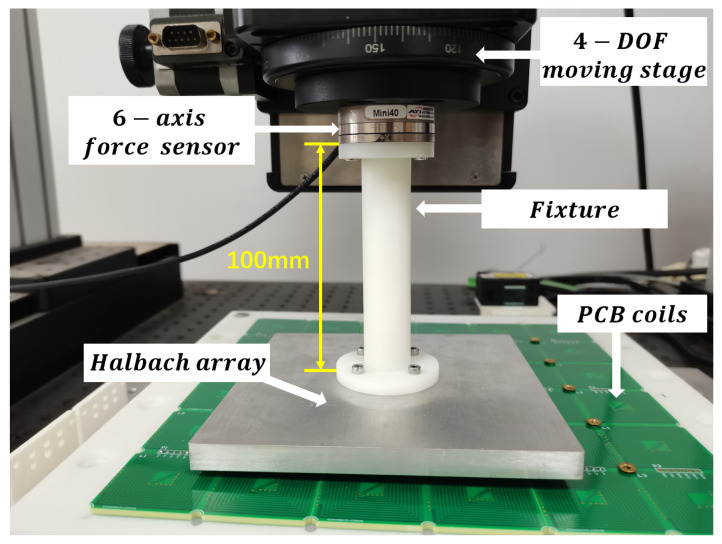
Experimental device for measuring force and torque.

To validate the accuracy of the proposed model, an experimental setup, depicted in Figure 10, is utilized for measurement and verification purposes. The setup consists of a four-degree-of-freedom motion platform capable of movement in the *x*, *y* and *z* directions, as well as rotation around the *z*-axis within the xy plane. Beneath the moving stage, a six-axis force and torque sensor is fixed to measure the forces and torques exerted on the translator. The connection between the Halbach array and the sensor is established through a 3D-printed fixture, with the fixture height set at 100 mm, to ensure torque measurement 100 mm above the Halbach array. For enhanced performance compared to traditional copper-wound coils, the system incorporates PCB coils that exhibit a higher current density. By applying a direct current (DC) of 1A to a single coil using a DC power supply, the current density can reach 5.4 A/mm${}^{2}$.

**Figure 11:**
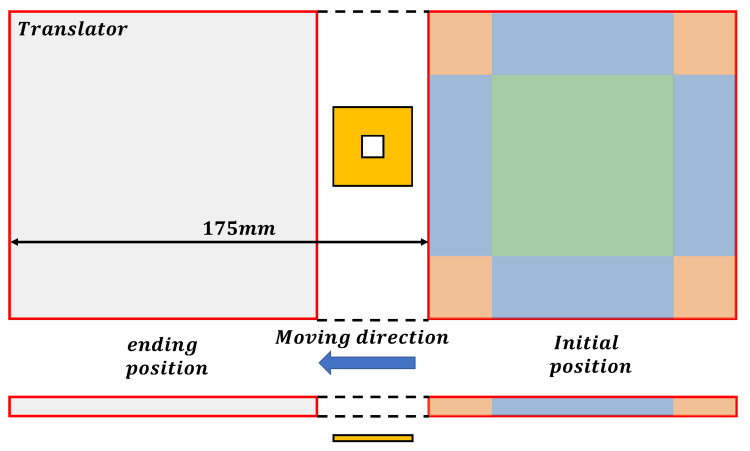
Force and torque computation on an offline program.

**Figure 12:**
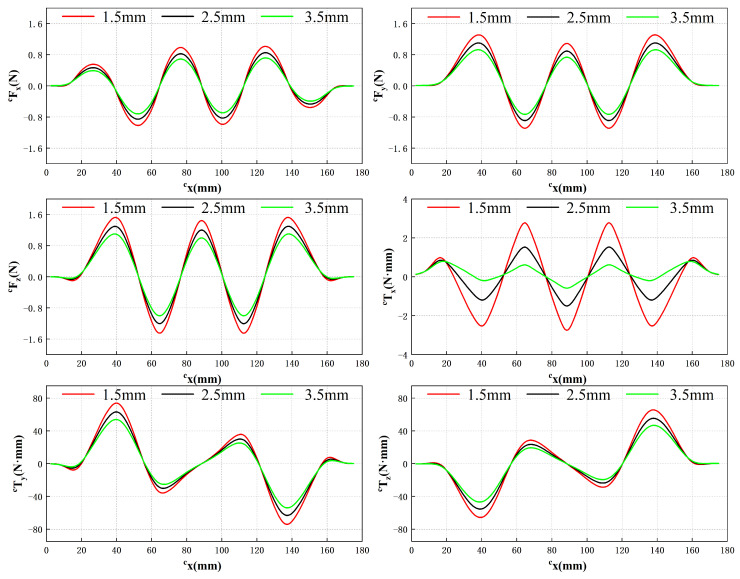
Force and torque acting on the translator under different air gaps when *y* = 6 mm.

During force measurement experiments, the protocol involves initially supplying 1A of current to a single coil using the DC power source. The four-degree-of-freedom moving stage is then controlled to transport the translator at a predetermined height, allowing the energized coil to sequentially pass through side region 2, the center region, and side region 4, as illustrated in Figure 11. The distance between the translator and the PCB coil is maintained at a specific value to simulate the suspension of the translator during normal system operation, representing the suspension height. This study selects three heights: 1.5 mm, 2.5 mm, and 3.5 mm. At each height, the force and torque acting on the translator are measured as the translator moves along *y* = 6 mm, while the coil traverses the three designated regions in sequence. The corresponding results are depicted in Figure 12.

In the global coordinate system, the ${}^{c}y$ coordinate of the translator remains constant, while the displacement in the ${}^{c}x$ direction is employed as the abscissa to illustrate variations in force and torque along the ${}^{c}x$-axis. The experimental measurements demonstrate that an increase in the air gap between the translator and the PCB coil leads to a reduction in the force and torque exerted on the translator. Regardless of the specific air gap value, the force and torque exhibit consistent trends with only variations in magnitude.

This behavior can be attributed to the fact that as the air gap widens, the coil moves farther away from the magnetic field region generated by the magnet array, thereby resulting in a decrease in the interaction force between the coil and the translator. Additionally, it is evident that at the initial and final stages of the translator’s motion, when the coil is positioned near the edge of the magnetic field, the force acting on the translator no longer follows a sinusoidal pattern but approaches zero. These outcomes align as expected based on the underlying principles of the system.

**Figure 13:**
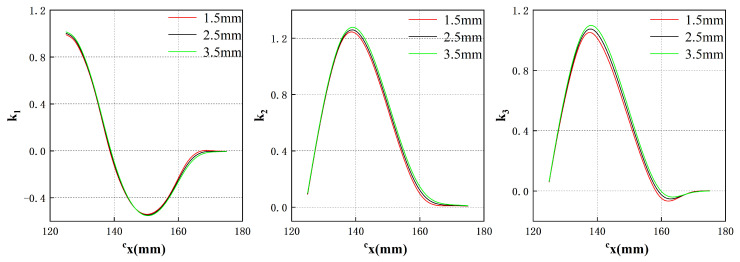
Three coefficients $k_{1}$, $k_{2}$ and $k_{3}$ under different air gaps.

Based on the results depicted in Figure 12 and utilizing Equation (8), three coefficients, identified as $k_{1}$, $k_{2}$ and $k_{3}$, can be determined to correspond to different air gap values, as illustrated in Figure 13.

**Figure 14:**
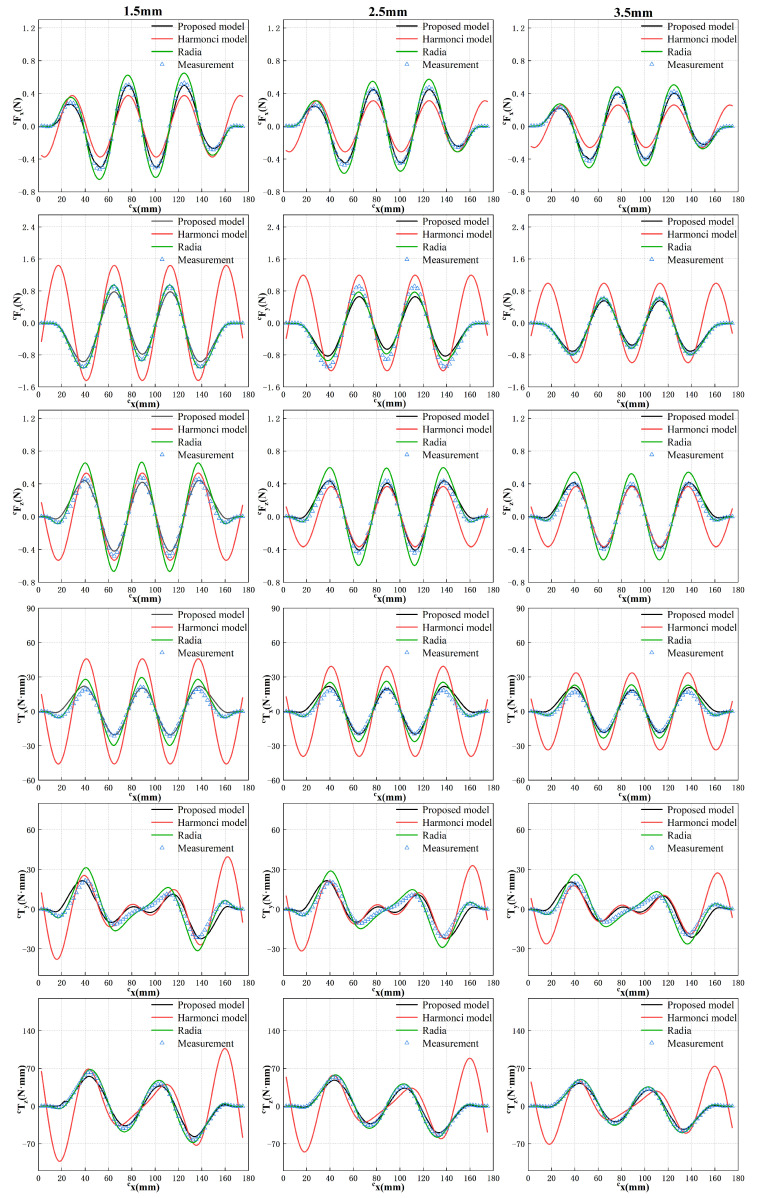
Comparison of calculation, simulation results and measurement results at *y* = 70 mm.

Once these coefficients are determined for a specific air gap, the force and torque acting on the translator at any point at that suspension height can be obtained. To validate the feasibility of this approach and the accuracy of the obtained *k* values, additional experiments are conducted. In these experiments, the three coefficients obtained are used to calculate the force and torque experienced by the translator at three different air gaps when *y* = 70 mm. While maintaining this specific ${}^{c}y$ coordinate, the translator is moved according to the motion sequence illustrated in Figure 11, with the coil passing through corner region 1, side region 1, and corner region 4 successively. Notably, the end effects occur when the coil is situated in these regions. The translator’s step distance is set to 2.5 mm, and the force and torque are measured at every 2.5 mm of movement. The proposed model, the traditional harmonic model, the result obtained by Radia and measurement results are compared, as shown in Figure 14.

Through a comprehensive comparison between the experimental results obtained from the proposed model and the actual values, a high level of consistency was observed. Furthermore, the simulation results obtained using the commercial software, Radia, further substantiate the accuracy of the proposed model. In the majority of cases, the proposed model exhibits closer proximity to the actual values compared to the simulation results. Therefore, the proposed model can be used instead of commercial software for calculation in practical applications. Notably, our proposed model exhibited significantly superior accuracy when compared to traditional harmonic models, particularly in capturing the intricate dynamics associated with end effects. The experimental findings convincingly demonstrate that our proposed approach outperforms traditional harmonic model methods, yielding an impressive enhancement in the calculation accuracy of magnetic force by 50.31% and torque by 70.65%.

Collectively, these experiments serve as compelling evidence to validate the effectiveness of our proposed model and the end-effects fitting technique. The confirmation obtained from this validation process instills confidence in the predictive capabilities of our model while substantiating its capacity to faithfully represent the intricate behavior of the planar motor across diverse operating conditions. These outcomes reinforce the significance and practical utility of the proposed model, which can be employed as a valuable tool in accurately predicting and analyzing the force and torque characteristics of planar motors.

### 5.2. Validation of the Solution of the Current Vector

**Figure 15:**
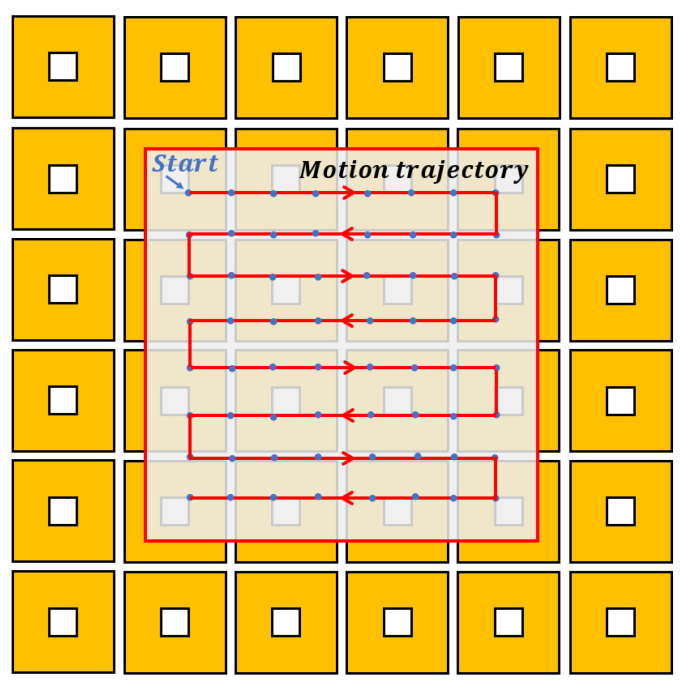
Test trajectory of the decoupling experiment.

To evaluate the decoupling effect of magnetic force and torque, a wrench vector ${}^{c}w$ acting on the translator is assumed. Specifically, $\left[\begin{array}{cccccc} 0\;N & 0\;N & 10\;N & 0\;N\;\cdot \;\mathrm{mm} & 0\;N\;\cdot \;\mathrm{mm} & 0\;N\;\cdot \;\mathrm{mm} \end{array}\right]$ at 64 points, corresponding to a force of 10 N acting along the *z*-axis and no torque applied. These sampling points are located on a specific trajectory in the 81 mm × 81 mm range as depicted in Figure 15. The current vector I at each point is calculated by solving the equations derived from the current–wrench transformation matrix, as described in Equation (21).

**Figure 16:**
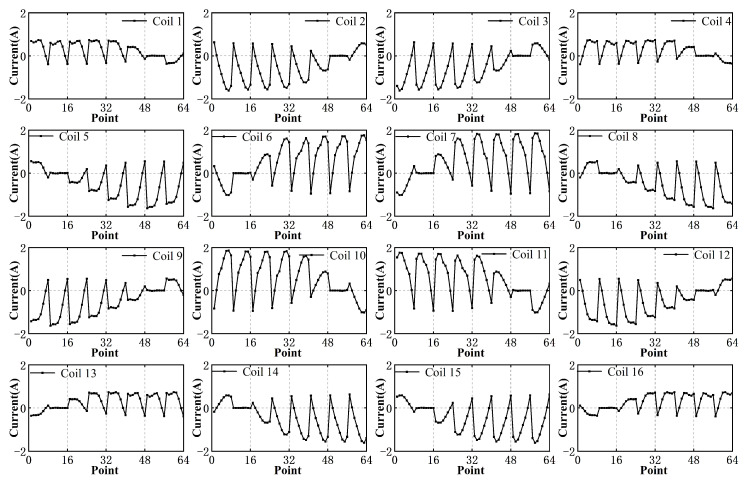
Current value of each coil.

The results are presented in Figure 16, which exhibit periodic changes due to the symmetry of the motion trajectory of the translator. Notably, the computed current values are reasonable and do not exceed 2 amps, thus indicating that these current values can be realized using a general amplifier.

**Figure 17:**
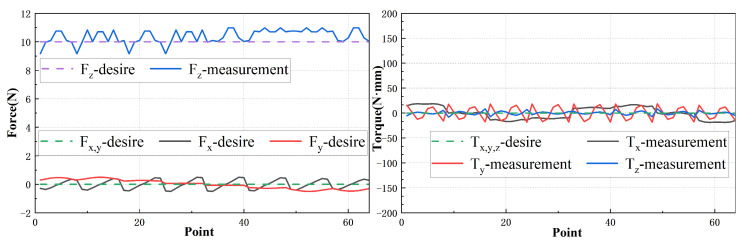
Force and torque solved by the obtained current vector.

In order to further validate the accuracy of the computed current values, a series of experiments are conducted. The measurement results are then compared to the corresponding desired results, as depicted in Figure 17. The measurement results exhibit close agreement with the desired results, confirming that the calculated current values are capable of providing sufficient force and torque to the translator. In general, our results demonstrate the effectiveness of our proposed model in accurately predicting the current required to achieve desired force and torque outputs, while also effectively decoupling these two parameters. These findings have practical implications for the design and control of planar motors, enabling precise and efficient operation.

## 6. Conclusions

This paper proposes a novel infinite expansion magnetically levitated planar motor that is based on printed circuit board (PCB) stator coils. In contrast to conventional magnetic levitation systems that utilize PCB coils, this design features smaller coil units, with each coil operating independently. The higher current density characteristic of the proposed design enables it to generate sufficient magnetic force, allowing for the realization of infinite expansion of the entire system. Furthermore, magnetic field modeling is conducted for both the stator coil and the 2D Halbach array structure utilized in the system. To accurately account for end effects during system motion, a simpler table lookup method is employed. A prototype of the testing principle is built, and experimental results are compared with traditional harmonic models, simulation results and measurement results, demonstrating excellent agreement.

Through the decoupling of force and torque, the current of each coil can be obtained. The experimental outcomes validate the feasibility and effectiveness of the proposed approach, which is more suitable for magnetically levitated planar motors with infinite expansion capability. The proposed model represents a valuable tool for accurately predicting and analyzing the force and torque characteristics of the system under different operating conditions. 

## Figures and Tables

**Table 1:** Parameter of the magnetically levitated planar motor.

Parameter	Value	Unit
Coil thickness $h_{s}$	2.5	mm
The hollow side length of coil $l_{1}$	9	mm
The single side width of coil $l_{2}$	12	mm
The side length of a square coil $l_{s}$	33	mm
Permanent magnet length $l_{m}$	16	mm
Permanent magnet width $w_{m}$	8	mm
Permanent magnet thickness $h_{m}$	8	mm
The pole pitch τ	24	mm
Magnet remanence $B_{r}$	1.28	T
Magnet field edge length $l_{center}$	38	mm

## Data Availability

Not applicable.
